# The Role of Circular RNAs in the Physiology and Pathology of the Mammalian Ovary

**DOI:** 10.3390/ijms232315204

**Published:** 2022-12-02

**Authors:** Jinbi Zhang, Caixia Wang, Chao Jia, Yi Zhang, Xinxin Qing, Yuge Zhang, Jingge Liu, Shiyong Xu, Zengxiang Pan

**Affiliations:** 1 College of Animal Science and Food Engineering, Jinling Institute of Technology, Nanjing 211169, China; 2 College of Animal Science and Technology, Nanjing Agricultural University, Nanjing 210095, China

**Keywords:** circRNAs, ovary, follicle development, atresia

## Abstract

Circular RNAs (circRNAs) are an abundant class of endogenous non-coding RNAs (ncRNAs) generated from exonic, intronic, or untranslated regions of protein-coding genes or intergenic regions. The diverse, stable, and specific expression patterns of circRNAs and their possible functions through cis/trans regulation and protein-coding mechanisms make circRNA a research hotspot in various biological and pathological processes. It also shows practical value as biomarkers, diagnostic indicators, and therapeutic targets. This review summarized the characteristics, classification, biogenesis and elimination, detection and confirmation, and functions of circRNAs. We focused on research advances circRNAs in the mammalian ovary under conditions including ovarian cancer, polycystic ovarian syndrome (PCOS), and maternal aging, as well as during reproductive status, including ovarian follicle development and atresia. The roles of circRNAs in high reproductive traits in domestic animals were also summarized. Finally, we outlined some obstructive factors and prospects to work with circRNA, aiming to provide insights into the functional research interests of circRNAs in the reproduction and gynecology areas.

## 1. Introduction

Circular RNAs (circRNAs) are a highly abundant class of endogenous non-coding RNAs (ncRNAs) discovered originally in RNA viruses [1] and then demonstrated ubiquitously in eukaryotic organisms in recent decades [2]. Studies suggested that circRNAs are primarily within 200–1000 nt in size (range from100 nt to over 4 kb) [3,4], and participate in multiple molecular processes in physiological and pathological conditions by posttranscriptional regulation of classical cell signaling pathways such as PI3K/Akt, Wnt/β-catenin, and RTK/Ras [5]. The fertility potential of the female is based on the development, growth, and final maturation and ovulation of ovarian follicles, which consist of an oocyte surrounded by granulosa cells (GCs) and theca cells (TCs). Follicular growth starts from primordial follicles, undergoes primary, secondary, preantral, and antral stages, and then continually grows during follicle recruitment, selection, and ovulation [6]. Under natural conditions, the mammalian follicle utilization rate is extremely low, as approximately 90% of follicles are removed from the ovary through a degenerative process known as atresia [7,8]. Dysregulation during follicle growth also leads to ovarian dysfunctions such as polycystic ovarian syndrome (PCOS) and ovarian cancer. Here, the characteristics, biogenesis and elimination, function, and detection of circRNA are summarized. The research advances of circRNAs in mammalian ovarian follicle development, atresia, and related dysregulation and diseases are reviewed.

## 2. Characteristics, Functions and Detection of CircRNA

### 2.1. Stability, Abundance, Conservation, and Specificity of CircRNAs

As the name implies, the most critical feature of circRNAs is their covalently closed loop structure without free 5′,3′ ends and poly(A) tail. As a result, circRNAs resist exonucleases (which require free ends) represented by RNase R, while still sensitive to endonucleases such as RNase A [9]. With such stability, circRNAs have remarkably longer half-life (20–48 h) than their full-length linear RNA counterparts (4–10 h) [10], and, therefore, have great potential as biomarkers.

Despite being widely expressed in most tissues, circRNAs showed a lower global abundance than their cognate mRNAs with some single-gene level exceptions, in which circRNAs can be 10-fold more abundant than their linear analogues [11,12]. Interestingly, although not completely confirmed, there seems to be a negative correlation between circRNA levels and cell division rate. Evidence announced an age-dependent accumulation of circRNA, which lead to their enrichment in brain tissue [13,14], and a division-driven ‘dilution’ of circRNA, which results in lower universal levels of circRNAs in cells with higher proliferative rates [15].

The evolutionary conservation of circRNAs has been proved by their sequences (originating from conserved exons/introns across eukaryotic species), formation manner, and even regulatory mechanisms [16,17]. Meanwhile, a remarkable cell/tissue and stage-specific expression pattern of circRNAs has been proved by numerous high-throughput studies, and thus makes them specific and reliable biomarkers [18]. The potential of circRNAs as biomarkers in diseases including cancer [5,19], cardiovascular [20], and ageing-related [21] conditions have been well summarized.

### 2.2. Classification, Biogenesis, and Degradation of CircRNAs

According to their distinct sequence composition, circRNAs are classified into three main types: exonic circRNAs (ecircRNAs); intronic circRNAs (ciRNAs); and exon–intron circRNAs (EIciRNAs). Note that three other classifications were also announced recently. tRNA intronic circRNAs (tricRNAs) were so far only reported in archaea and Drosophilid species, in which tRNA introns are highly conserved [22]. Interior circRNAs (i-circRNAs) originate from inside exons, introns, and intergenic transcripts under the trigger of AC/CT motif [23]. Moreover, a small group of circRNAs that are synthesized in an antisense orientation, termed antisense circRNA, were revealed by high-throughput analysis [24].

The essential event for most circRNA biogenesis is the back-splicing process that ligates a downstream 5′ splice donor site with an upstream 3′ splice acceptor site to form a closed-loop structure. The detailed circularization may be induced through several non-exclusive pathways, including lariat-driven circularization (exon skipping), direct back splicing (intron-pairing driven circularization), and spatial proximity due to binding and interaction of RNA-binding proteins (RBPs). The varied biogenesis processes were represented nicely by the schematic diagrams in reviews of Huang [25], Tran [26], and Zang [27].

The typical RNA degradation pathways are inapplicable for circRNAs as they have no free ends. Recent studies found that circRNA degradation can be initiated by N^6^-methylation of adenosine (m^6^A) recruited endonucleases [28] and the release of extracellular vesicles or microbubbles such as exosomes [29,30]. Moreover, posttranscriptional small RNA-mediated silencing of circRNAs was also identified [31], and related artificial shRNA/siRNA-based systems have been well used for circRNA loss-of-function studies [2,32].

### 2.3. Molecular Functions of CircRNAs

In the cytoplasm, the most straightforward function of circRNA EOC s is based on the complementary binding of miRNAs as sponges, which reduces active miRNA and consequently regulates the expression of its target genes [33]. In other words, both circRNA and mRNA may have complementary sites of particular miRNAs. Thus, they can compete for the binding of the same miRNA. In this case, circRNAs are considered competitive endogenous RNA (ceRNA) in the circRNA-miRNA-mRNA networks. In addition, based on the specific circRNA–protein interaction, circRNAs have also been shown to interact with RBPs, act as a direct protein sponge/sequester, or constitute a scaffold or decoy to facilitate other protein interactions [34]. Moreover, although not yet proved in mammals, some circRNAs containing open reading frames (ORF) showed translation potential [35]. The circRNAs translation mechanism initiated by internal ribosome entry sites (IRESs) and m6A-induced ribosome engagement sites (MIRESs) was reviewed by Anne-Catherine, et al. [36]. On one hand, the possible proteins or peptides products of circRNAs may have their function per se, on the other hand, synthetic circRNAs can be used as a potent tool for durable protein production in vivo [37].

ciRNAs located in the nucleus may have diverse functions in the cis-regulation of genes. The formation of ecRNAs that are derived from exons may compete with the selective splicing of their precursor mRNAs (pre-mRNAs), which may lead to reduced levels of linear mRNAs and changes in their composition due to the missing of specific exons [38,39]. On the contrary, some circRNAs may promote the transcription of corresponding genes by interacting with the Pol II complex [40]. Currently, more functions of circRNAs are continuously being revealed.

### 2.4. Detection and Confirmation of CircRNAs

The detection, validation, and quantification of circRNAs is somehow analogous to studying other types of coding or non-coding RNAs. The main change is to separate circRNA from other RNA species as the majority of their sequence that is generated from the same host gene is shared. The combination of high-throughput sequencing and bioinformatic technology is no doubt the most effective way to explore novel circRNAs. General total RNA library can be used for circRNA identification/prediction with tools such as CircRNA finder [41], MapSplice [42], CIRI [43], and CIRCexplorer [44] based on spliced alignment algorithms. Further, circRNAs can be much enriched using rRNA-depleted and RNase R-treated libraries, which, however, carries with it the risk of losing large molecular weight circRNAs [45].

The hybridization-based assays are reliable for the validation of known circRNAs, and PCR-based methods can be used for quantification. Northern blot was considered the gold standard for confirming the size and configuration of circRNAs but was limited by its high requirement of RNA sample amount and time-consuming steps. For in situ detection of circRNAs, fluorescence in situ hybridization (FISH) with probes specific for the back-splice junction sites reveals their subcellular localization [46]. For batch targeted analysis, circRNA microarrays, which are commercially available from Arraystar and CapitalBio, etc., allow the identification of circRNAs expression levels on a large scale with a higher efficiency [47]. Compared to hybridization-based methods, PCR-based approaches are much more sensitive and can quantitate circRNA variation at the femtomolar level. An RNase R treatment must be added before cDNA synthesis, and primers focused on the specific splice junction must be used. In addition, adequate internal controls (foreign RNA spike-in) can be used to eliminate the RNA sample composition change caused by RNAse R treatment, and Sanger sequencing are necessary to guarantee the actual existence of circRNA. Furthermore, related methods, such as reverse transcription-droplet digital polymerase chain reaction (RT-ddPCR) [48] and Lexo-circSeq [49] are emerging rapidly.

The existing gain and loss of function methods used for gene function study can also be applied for overexpression and depletion of specific circRNA with certain adjustments. When applying RNAi-mediated degradation with small interfering RNA (siRNA) or short hairpin RNA (shRNA), target the back-splicing junction to achieve a circRNA-specific knockdown effect. Plasmids containing circRNA-producing exons and their flanking intronic sequences can be used to introduce circRNAs to cells by transfection. Detailed experimental methods for circRNAs validation were well reviewed and evaluated by Li et al. [50].

## 3. CircRNAs in Mammalian Ovaries

**Figure 1:**
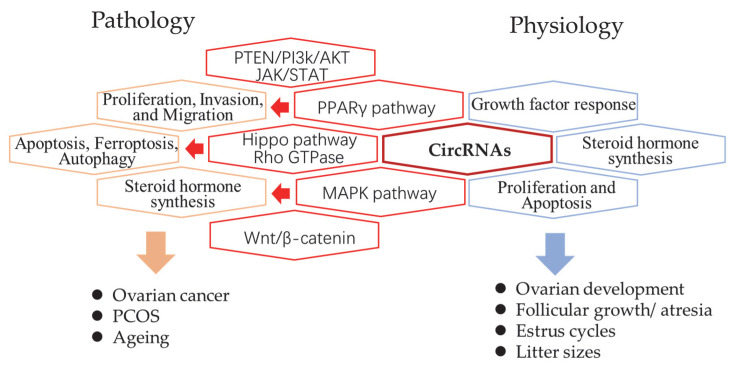
The summary of circRNAs related pathways in pathology and physiology processes of the mammalian ovary. The revealed circRNA-related signaling pathways (red hexagon) and their related biological processes in pathology (orange hexagon) and physiology (blue hexagon).

The genome-wide profiles of ovarian circRNAs were mainly reported in humans, mice, pigs, and goats. In humans, circRNA studies have been focused on pathological examination of ovarian cancer, PCOS, and ageing. Luckily, studies in animals provided more knowledge regarding ovary growth, changes in estrus, as well as follicle development, and atresia. Comparisons between different reproductive performances and breeds were also reported (Figure 1). Here, we reviewed the global studies of each field first and summarized the proven function of individual circRNAs in Table 1.

### 3.1. CircRNAs in Ovarian Cancer

CircRNAs in ovarian dysfunction attracted close attention due to their tight interaction with miRNAs. In 2015, a comprehensive assessment compared circRNA levels across several normal and cancerous tissues, including ovarian cancer, and discovered a global reduction in circular RNA abundance in cancer compared to normal tissues, therefore suggesting a negative correlation between circular RNA abundance and cell proliferation [15]. Ning et al. also performed circRNA-sequencing in epithelial ovarian cancer (EOC) and normal ovarian tissues and identified 4388 differently expressed circRNAs [51]. Almost simultaneously, Teng et al. analyzed circRNA expression profiles in EOC and normal ovarian tissues, in which the expressions of 5551 circRNAs were differentially expressed [52]. Gao et al. sequenced and compared circRNA in high-grade serous ovarian cancer (HGSOC) specimens and normal ovarian tissues. Among 710 differentially expressed circRNAs, circRNA1656 was confirmed down-regulated in HGSOC tissues and ovarian cancer cell lines [53]. Furthermore, Zhao et al. investigated the expression of circRNAs in paired cisplatin-sensitive and cisplatin-resistant tissues of ovarian cancer by microarray analysis and reported 339 aberrantly expressed circRNAs [54]. Cdr1as was proven to sensitize ovarian cancer to cisplatin by regulating the miR-1270/SCAI axis. Based on these high-throughput studies, a detailed functional analysis in single-circRNA level was reported in continuance, which showed great potential as biomarkers for ovarian cancer.

### 3.2. CircRNAs in PCOS

PCOS is the most common endocrine disorder in women of reproductive age. To reveal the functions of circRNAs in the development of PCOS, circRNA profiles from cumulus cells and follicle fluid were assessed, respectively. Che et al. determined 311 increased and 721 decreased circRNAs in cumulus cells from PCOS compared to control participants who underwent IVF using microarray [55]. With these data, Li et al. further combined data of microRNA and mRNA in PCOS to predict circRNAs which may serve as RBP regulators or miRNA sponges [56]. This study conducted a weighted correlation network analysis (WGCNA) to mine PCOS-associated circRNA-miRNA-gene networks and circRNA-RNA binding protein (RBP) networks. Moreover, Wang et al. performed a delicate study by sequencing ribosomal RNA-depleted total RNA from exosomes of follicle fluids. They identified 167 up-regulated and 245 down-regulated circRNAs in PCOS patients [57].

### 3.3. CircRNAs during Maternal Ageing

The decline of female reproductive capacity with age, termed ovarian senescence, results in a gradual reduction in the quantity and quality of oocytes. Cheng et al. first compared circRNAs in GCs from in vitro fertilization (IVF) patients with young (≤30) and advanced (≥38) ages using human circRNA microarrays. This study revealed 46 up-regulated and 11 down-regulated circRNAs in aged samples. Later, Cai et al. compared circRNA expression profiles between healthy ovarian cortex from young (25–28) and ageing (44–46) groups and identified 194 up-regulated and 207 down-regulated circRNAs enriched in oxidation-reduction, steroid hormone biosynthesis, and insulin secretion pathways, during ageing [58].

### 3.4. CircRNAs and Ovary Development

CircRNA profiles during ovarian development, estrus cycles, and follicular growth were explored mainly using large animals such as pigs and goats. CircRNA landscape in adult and neonatal ovaries was first examined and compared in mouse ovarian tissue using high-throughput sequencing. Estrogen signaling was found to be the most significant pathway that up-regulates in adult ovaries [59]. In pigs, more specifically, ovarian circRNA profiles at three developmental stages (0, 30, and 240 days after birth) were identified and compared with other eight tissues (heart, liver, spleen, lung, kidney, testis, skeletal muscle, and fat). This study revealed ovary-specific/enhanced circRNAs and provided valuable resources for ovarian circRNA study [60]. In addition, the profiles of ovarian circRNAs across pre-, in-, and post-pubertal stages were reported. The study identified 631 stage-specific circRNAs generated from genes involved in steroid biosynthesis, progesterone-mediated oocyte maturation, and autophagy [61].

Regarding the estrus cycle, Liu et al. analyzed the circRNA profiles of Yunshang black goat ovarian tissues among high and low fecundity groups in the follicular phase and luteal phase, and conclude that circRNAs play a key role in both the prolificacy trait and transformation of the follicular phase to the luteal phase in the estrus cycle [62]. At the follicle level, Xu et al. reported 290 differentially expressed circRNAs between large (diameter > 4 mm) and small (diameter < 4 mm) follicles in Dazu black goats. This study also simultaneously generated profiles of mRNAs, long non-coding RNAs (lncRNAs), and microRNAs (miRNAs), creating a good start and helpful reference for integrated ncRNA study during follicle development [63]. To explore the roles of circRNA in growth factors response, Fu et al. [64] profiled circRNAs of bovine cumulus cells treated with or without growth factors (bone morphogenetic protein 15 (BMP15), growth differentiation factor 9 (GDF9), and BMP15+GDF9). This study suggested that GDF9 induced a more significant circRNA shift than BMP15, and BMP15 may play a role in assisting GDF9. Changed circRNAs were involved in pathways, including thyroid hormone signaling ubiquinone and terpenoid-quinones, which affected the proliferation and apoptosis of CCs.

### 3.5. CircRNAs and Follicular Atresia

circRNA profiles in healthy and atretic antral follicles were first deep sequenced by Guo et al., and 192 circRNAs were reported to be differentially expressed during the atresia process [65]. Based on this study, detailed functions of circRNAs serving as miRNA sponges in the connective tissue growth factor (CTGF) regulatory pathway [65], inhibin–activin balance [66], and cell viability [67] have been reported. It is widely accepted that GCs play a significant role in the follicular development and atresia processes, thus determining the fate of follicles [68]. Therefore, Meng et al. performed a more specific study to profile circRNAs generated from porcine granulosa cells isolated from healthy atretic antral follicles [69], which is a perfect supplement and advancement to the earlier research. This study further confirmed circRNA functions in oxidative stress inhibition and cell apoptosis pathways.

### 3.6. CircRNA and High Reproductive Traits

To explore the circRNA functions in reproductive performance, the circRNA function in litter size was investigated in pigs, goats, and sheep. In pigs, circRNA profiles of ovaries from large and small litter sizes groups were performed by Xu et al. [70], and 56 down-regulated and 54 up-regulated circRNAs were observed in the large litter sizes group. Parallelly, a similar study of ovaries from MeiShan (local breed with large litter) and Large White pigs was performed and revealed 37 up-regulated and 48 down-regulated circRNAs [71]. The pre-ovulatory follicles of the Boer goat and Macheng black goat, which is highly fertile with a twin and multiparous lamb rate of 70%, were compared [72]. This study not only examined goat ovarian circRNA profile for the first time but also identified 37 differentially expressed circRNAs in high litter size breeds. A more delicate analysis was performed in ovarian tissues from both follicular and luteal phases of Harper sheep that were either consecutive monotocous or polytomous. Totals of 183 and 34 differentially expressed circRNAs were identified in h follicular and luteal phases, respectively, and TGF-β and thyroid hormone signaling were highlighted to affect the litter size through circRNAs [73]. However, all these studies suggested that in the ovary, the number of circRNAs that varies between breeds or reproductive performance is relatively low. During the follicle cycle, destined ovarian follicles grow rapidly, which is based on the rapid division of granulosa cells. Therefore, such observations agree with the speculation of a negative correlation between circRNA levels and cell division rate in cancer studies. Moreover, an interesting study in rats revealed potential functions of circRNAs in continuous light-induced ovarian dysfunction, which provided novel clues of circRNA shift in response to temporary environmental changes [74].

**Table 1:** Circular RNAs and their role in the different ovaries.

Species	Tissue	CircRNA	Target miRNA/Gene/Protein	Function	Ref.
human	OC	Cdr1as	miR-1270/SCAI	sensitizes ovarian cancer to cisplatin	[54]
		circ-ITCH	miR-145/RASA1	inhibit tumour progression	[75]
		has_circ_0051240	miR-637/KLK4	suppresses cell proliferation, migration, and invasion	[76]
		circEPSTI1	miR-942	inhibit cell growth and invasion, induces apoptosis	[77]
		circRNA CDR1	miR-135b-5p/HIF1AN	decreasing the occurrence and progression of ovarian cancer	[78]
		circLARP4		down-regulated in cancerous ovarian cells	[79]
		hsa_circ_0007444	miR-23a-3p/DICER1		[80]
		circPLEKHM3	miR-320a/SMG1	exacerbated the effect of curcumin on ovarian cancer cell proliferation and apoptosis, as well as the anti-tumour effect	[81]
		circABCB10	miR-1271	promotes cell proliferation and invasion but inhibits apoptosis	[82]
		circRNA1656	miR-1301-3p/miR-4660-SIRT3	down-regulated in HGSOC	[53]
		circ-CSPP1	miR-1236-3p	promotes proliferation, invasion, and migration	[83]
		has-circ-001567		promotes cell proliferation and invasion	[84]
		circ-SMAD7	KLF6	promotes cell proliferation and invasion	[85]
		circ_0025033	miR-184/LSM4	promotes the progression of ovarian cancer	[86]
		circHIPK3		related to cell growth, migration, and apoptosis	[52]
	PCOS	circ_0023942	CDK-4	inhibit granulosa cell proliferation	[87]
		circ_0043533	miR-1179	related to Bcl-2, CDK2, and Cyclin D1	[88]
		circ_RANBP9	miRNA-136-5p/XIAP	exacerbates POS	[89]
		circASPH	miR-375/MAP2K6	promotes cells proliferation	[90]
		circRHBG	miR-515/SLC7A11	knockdown of circRHBG promotes ferroptosis in PCOS	[91]
		circ_0005925	miR-324-3p/MAP2K6	Promotes Granulosa Cell Growth	[92]
		circ_0043532	miR-182/SGK3	promote cell proliferation	[93]
	ovary	circDDX10		ovarian aging	[58]
	KGN	circUSP36	PTBP1/NEDD4L	enhance autophagic granulosa cell death	[94]
	GCs	circDDX10		affecting the proliferation and apoptosis and steroid hormone synthesis	[95]
Pig	ovary	circ-TCP11	miR-183	associated with swine litter size	[70]
	ovary	circSCIN	miR-133, miR-148a/b	affecting estrogen secretion	[71]
	GCs	ssc-circINHA-001	miR-214-5p, miR-7144-3p, miR-9830-5p/INHBA	mediated Inhibin–Activin balance	[66]
	GCs	circSLC41A1	miR-9820-5p/SRSF1	resists porcine granulosa cell apoptosis and follicular atresia	[67]
	GCs	circ-ANKHD1	miR-27a-3p/SFRP1	decreased the cell apoptosis rates	[96]
Bovine	GCs	circ_n/a_75	miR-339a	growth factor response	[64]
		circ_n/a_303	miR-2400 and miR-30c		[64]
Goat	follicles	chi_circ_0008219	miR-34c-5p, miR-483, miR-1468-3p	higher fecundity rate	[72]
Mouse	GCs	circEGFR	miR-125a-3p/CYP19A1	promoted granulosa cell apoptosis	[59]

## 4. Conclusions

circRNA-mediated regulation in the ovary has drawn more and more attention recently. Unlike miRNAs, which are well examined in follicular development, atresia, and ovarian disorders [97], the biological functions of circRNAs in the ovary are still largely unknown. The reasons include, but are not limited to:

(1) The detection and validation of circRNAs are less straightforward and accurate quantification and manipulation of circRNAs are more time-consuming and are more likely to be affected by the linear mRNA encoded by the same gene;
(2) The conservation of circRNA among species is relatively low, which makes it difficult to make comparisons and transfer discovery of one species to another;
(3) The subcellular location and molecular function of circRNAs are varied, which adds complexity to reveal the functional networks of circRNAs. However, precisely because of their versatile and unique structure, circRNAs became a research hotspot of great potential.

In the ovarian pathology area, circRNAs show tumor-promoting properties by either increase OC cell proliferation, invasion and migration through PTEN/PI3k/AKT, JAK/STAT, and MAPK signalings or indirectly exert the suppression mechanisms, such as apoptosis, ferroptosis and autophagy pathways through PPARγ, Hippo or Rho GTPase signalings. The involvement of circRNAs also affect ovarian function by contributing in oxidation–reduction and steroid hormone biosynthesis processes. The highly stable nature and cell specificity of circRNAs makes them promising biomarkers for disease as they were detectable in whole blood, plasma, and platelets [98] when released into the bloodstream from damaged or diseased cells over time.

In the female reproduction area, the following aspects may attract more attention in future studies:

(1) circRNAs involved regulatory networks in critical pathways such as steroid hormone biosynthesis, programmed cell death (apoptosis, autophagy, ferroptosis), and oxidative stress response during ovarian physiological processes;
(2) circRNAs involved in tumorigenic or suppressive pathways which can be used as therapeutic targets;
(3) circRNA serving as stable diagnostic and prognostic biomarkers to assess the reproductive status, disease, and applications in assisted reproductive technology;
(4) The roles of exosomal circRNAs in oocyte-granulosa-thecal cell communication;
(5) Identification and functional studies of proteins or peptides products of circRNAs;
(6) Development of practical and effective techniques for quantitative detection of circRNAs. Although our understanding and application of circRNAs in the mammalian ovary are still in the initial stage, these unique molecules hold immense potential for further research and will pave new avenues for the field.

## Data Availability

Not applicable.
